# Evaluation of color stability and surface roughness of smart monochromatic resin composite in comparison to universal resin composites after immersion in staining solutions

**DOI:** 10.1186/s12903-025-06555-5

**Published:** 2025-07-19

**Authors:** Ammar Shawkat Abdul kareem, Wegdan Mohamed Abdel-Fattah, Marihan Ibrahim Lotfy El Gayar

**Affiliations:** Department of Conservative Dentistry, Faculty of Dentistry, Alexandria University, Champollion St, Azarita, 21527 Alexandria Egypt

**Keywords:** Color stability, CIEDE2000, Smart monochromatic resin composite (omnichroma), Surface roughness, Thermocycling

## Abstract

**Background:**

The color stability and surface roughness of resin composites are the primary factors influencing the qualities of restorative materials. This study aimed to assess the color stability and surface roughness of a smart monochromatic resin composite submerged in various staining solutions.

**Materials and methods:**

Ninety-nine disc samples of composite resins (8 mm in diameter and 2 mm in thickness) were prepared and divided into three groups (*n* = 33): Group A, Omnichroma (Tokuyama Dental, Tokyo, Japan); Group B, Neo Spectra ST HV (Dentsply, Konstanz, Germany); and Group C, Filtek Z350XT (3MESPE) composite resins. The samples were assessed for color stability (M) via a Vita Easyshade spectrophotometer and surface roughness (Ra) with a profilometer before and after thermocycling for a total of 1000 cycles at 5°/55°C with a 15-second dwell time. Each group was subdivided into three subgroups according to immersion solution (*n* = 11): Subgroup I was submerged in distilled water as a control, Subgroup II was submerged in tea, and Subgroup III was submerged in coffee. Color assessment was performed after immersion periods of T1: 7 days, T2: 15 days, and T3: 30 days with a Vita Easyshade spectrophotometer. A color change (∆E00) was obtained via the CIEDE2000 color difference formula. Surface roughness was assessed with a profilometer after 30 days of submersion in the staining solutions.

**Results:**

Among all the samples in the study groups, Omnichroma had the lowest color change value for all the immersion solutions at different time intervals (*p* < 0.001). The surface roughness of Omnichroma significantly differed after thermocycling (*P* = 0.075). However, Omnichroma had the least surface roughness compared with Neo Spectra ST HV (*P* = 0.026) and Filtek Z350XT (*P* = 0.024).

**Conclusion:**

The color change and surface roughness of the supra-nanofilled (Omnichroma) resin-based composite were inferior to those of the nanohybrid (Neo Spectra) and nanofilled (Filtek Z350XT) composites.

**Supplementary Information:**

The online version contains supplementary material available at 10.1186/s12903-025-06555-5.

## Introduction

Color stability and surface properties are crucial for successful restorative materials, but discoloration is a common cause of restoration failure, especially when these materials are subjected to the oral environment. Three categories of discoloration are identified: external discoloration resulting from accumulated plaque and surface staining, surface or subsurface discoloration resulting from superficial deterioration or dye infiltration, and intrinsic discoloration caused by physicochemical processes within the resinous matrix in the superficial and inner layers of the material [1].

The color change of resin composites can be influenced by multiple factors, such as the composition and structure of the resin composite, the features of the filler particles used, the degree of polymerization, and the amount of water absorbed [2]. Composite resins might change color when exposed to staining solutions, such as coffee, cola, and tea. These beverages can affect restoration quality and influence the physical and aesthetic properties of composite resins [3].

Accurate color matching of restorations to natural teeth is crucial for patient satisfaction and restoration acceptability. Shade matching may be achieved visually with tooth-shaped shade tabs or instrumentally via color measurement instruments. The VITA-3D Master improves shade matching by including the color spectrum of human teeth, making it more effective [4].

Surface roughness is often determined by the Ra (roughness average) value via the use of profilometry to evaluate surface quality. Various methods have been used to evaluate the roughness of a surface. The technologies used include confocal laser scanning microscopy (CLSM), laser reflectivity, contact stylus tracing, scanning electron microscopy (SEM), transmission electron microscopy (TEM), and atomic force microscopy (AFM) [5, 6].

The Smart Monochromatic Composite is a widely used shade-matching composite that employs smart chromatic technology without additional dyes or pigments. Unlike traditional fillers, it creates reddish-to-yellowish structural hues. Reducing the number of color selections available to match different chromatic ranges of teeth facilitates better shade matching [7].

This in vitro study aimed to evaluate and compare the color stability and surface roughness of different composite materials: smart monochromatic (single shade) and multishade resin composites. The null hypothesis of this study was that there would be no statistically significant differences in the color stability and surface roughness of supra-nanofilled resin composite before and after thermocycling and immersion in various staining solutions compared with those of nanohybrid and nanofilled resin composites.

## Methods

### Specimen preparation

Ninety-nine composite resin disc samples were prepared from three restorative composites and then divided into thirty-three samples for each group of materials: group A: Omnichroma (Tokuyama Dental, Tokyo, Japan); group B: Neo Spectra ST HV, shade A3 (Dentsply, Konstanz, Germany); and group C: Filtek Z350XT, shade A3 (3MESPE) [Table 1]. The samples were fabricated via a Teflon mold with an 8 mm diameter and 2 mm thickness by condensing the material into the mold [8]. All composite disc samples were polymerized via an LED light curing unit (Premium Plus light cure C02-D, Premium Plus-UK, England) with an intensity of 1,200 mW/cm² for 20 s in continuous curing mode [9].

**Table 1:** Composition of the restorative materials employed in the current study

Trade name	fillere type and size	Resin matrix	Filler volume	Filler weight	Shade	Manufacture	Product number
Omnichroma	Uniform-size supra-nanospherical fillers, especially spherical silica and zirconia, with a size of 260 nm.	TEGDMA UDMA	68%	79%	Single shade	Tokuyama Dental, Tokyo, Japan.	2881
Filtek Z350 XT	Nanofilled, non-agglomerated 20 nm silica filler and 4 to 11 nm zirconia filler, and aggregated zirconia/silica cluster filler consisting of 20 nm silica and 4 to 11 nm zirconia particles.	TEGDM, UDMA, Bis-GMA, PEGDM, and Bis-EMA	63.3%	78.5%	A3	3 M ESPE, Min- nesota, USA	10,062,885
Neo Spectra ST HV	Nano-hybrid prepolymerized spherical fillers consist of barium glass fillers of 15 μm and 0.6 μm, along with 0.6 μm ytterbium fluoride and 10 nm silicon dioxide nanofillers.	Methacrylatemodified polysiloxane (organically modified ceramic) dimethacrylate resins, ethyl-4 (dimethylamino) benzoate, and bis (4methylphenyl) iodonium hexafluorophosphate.	60–62%	78–80%	A3	Dentsply Sirona, DE, USA	2,303,000,656

*Abbreviations: Bis-GMA: bisphenol A-glycidyl methacrylate, UDMA: urethane dimethacrylate, TEGDMA: triethylene glycol dimethacrylate, Bis-EMA: bisphenol A polyethylene glycol diether dimethacrylate, and PEGDMA: polyethylene glycol dimethacrylate

The samples were finished for fifteen seconds [10] utilizing a multifluted tungsten carbide finishing bur (VERDENT, Poland) coupled to a high-speed handpiece at 200,000 rpm with a water coolant spray [11]. Afterward, polishing was applied via Sof-Lex Discs fine grit and superfine grit, along with a two-step Sof-Lex spiral wheel polishing system (3 M ESPE, St. Paul, MN, USA), which included a pre-polishing spiral (beige) and a polishing spiral (pink). A low-speed handpiece without a water coolant for 15 s was employed at medium speed (approximately 10,000 rpm), sprayed with water, and allowed to dry [12]. After finishing and polishing, the samples were subjected to ultrasonic cleaning with distilled water for 1 min and dried in oil-free air [13]. Then, all the samples were kept in distilled water at 37 °C for 24 h to facilitate rehydration and full polymerization [14].

### Color and surface roughness measurements (baseline)

A Vita Easyshade Compact spectrophotometer (Vita Zahnfabrik, Bad Sackingen, Germany) was used to perform the first color measurements (baseline) (M1) of the composite specimen discs performed inside a custom-made viewing booth under the same environmental conditions: TLD 95/65 (ISO 3668) color temperature of 6,500 K light conditions with a very high-quality daylight simulator [fluorescent tubes] with a black background. The probe was held perpendicular to the sample, and the results were displayed on an LED screen. The spectrophotometer was equipped with a fiber optic probe assembly for lighting and reception of the reflected light [15]. The CIEDE2000 formula was employed to compute the color difference (ΔE00) among all groups in different composite samples (discs):

This formula is suitable for examining weighting functions (SL׳, SC׳, and SH׳), parametric factors (KL, KC, and KH), and variations in lightness, chroma, and hue (ΔL, ΔC, and ΔH, respectively), potentially improving color match and visual assessments. The computation is explained in the following equation [16]:

$$\Delta {E_{oo}}\, = \,{\left[ {{{\left( {\frac{{\Delta {L^\prime }}}{{{K_L}{S_L}}}} \right)}^2}\, + \,{{\left( {\frac{{\Delta {C^\prime }}}{{{K_C}{S_C}}}} \right)}^2}\, + \,{{\left( {\frac{{\Delta {H^\prime }}}{{{K_H}{S_H}}}} \right)}^2}\, + \,{R_T}\,\left( {\frac{{\Delta {C^\prime }}}{{{K_C}{S_C}}}} \right)\,\left( {\frac{{\Delta {H^\prime }}}{{{K_H}{S_H}}}} \right)} \right]^{1/2}}$$

The surface roughness measurements (baseline) (Ra)1 were obtained via a contact stylus profilometer (Marsurf PS10; MAHR, Germany). The average roughness was assessed at various surface locations with a cutoff value of 0.8 mm, a speed of 0.5 mm/s, and a measurement length of 1.5 mm. The sample was measured at three different points, and the average surface roughness (Ra) of these three readings was recorded to identify the surface characteristics [17].

### Thermocycling process

The samples of each composite type were thermocycled for 1000 cycles, which simulated approximately 1–2 months in the oral environment. Each cycle included a dwell time of 15 s of immersion in the hot path at 55 °C ± 1 and then immersion in the cold path at 5 °C ± 1, with a 5 s delay between paths. After thermocycling, new color measurements (M)^2^ and surface roughness measurements (Ra)^2^ were performed [18].

### Immersion in different staining solutions

After thermocycling, the samples were submerged in distilled water, tea, and coffee for 3 h daily for 30 days [19]. After each round of staining, the samples were washed, dried, and maintained in distilled water at 37 °C inside an incubator [14]. Staining solutions were replaced every two days to prevent bacterial or yeast contamination [20]. Color measurements (M)³ of the samples immersed in the staining solutions were performed at T1: 7 days, T2: 15 days, and T3: 30 days, whereas roughness measurements (Ra)³ were taken on each sample via a profilometer after only 30 days of storage [21]. The tea solution was prepared by submerging a teabag (Lipton, yellow label, Unilever brand, Egypt) in 150 ml of boiling water for 5 min [22], while the coffee solution (Nescafe Classic, Nestle, Egypt) was made by mixing 15 gm of instant coffee powder into 200 ml of boiling water, which was measured via a digital scale [23].

### Statistical analysis

The Shapiro‒Wilk test was applied to assess the normality of the distributions of all the variables. The data were analyzed as the means and standard deviations (SDs) via one-way ANOVA and Tukey’s post hoc test with Bonferroni adjustment to assess the effects of thermocycling on color change and surface roughness between the groups, whereas a paired t-test was used to assess variations in surface roughness after thermocycling. Two-way ANOVA was used to examine the influence of the material, immersion solution, and their interaction on the surface roughness. Two-way multiple measurement analysis of variance was applied to estimate the influences of the material, immersion solution, immersion duration, and interaction among these variables. A post hoc analysis was performed via Bonferroni adjustment to correct for Type I errors. All tests were two-tailed, with a significance threshold of a p-value < 0.05. The data were examined via IBM SPSS version 25 (Armonk, USA).

## Results

### Color analysis

**Fig. 1:**
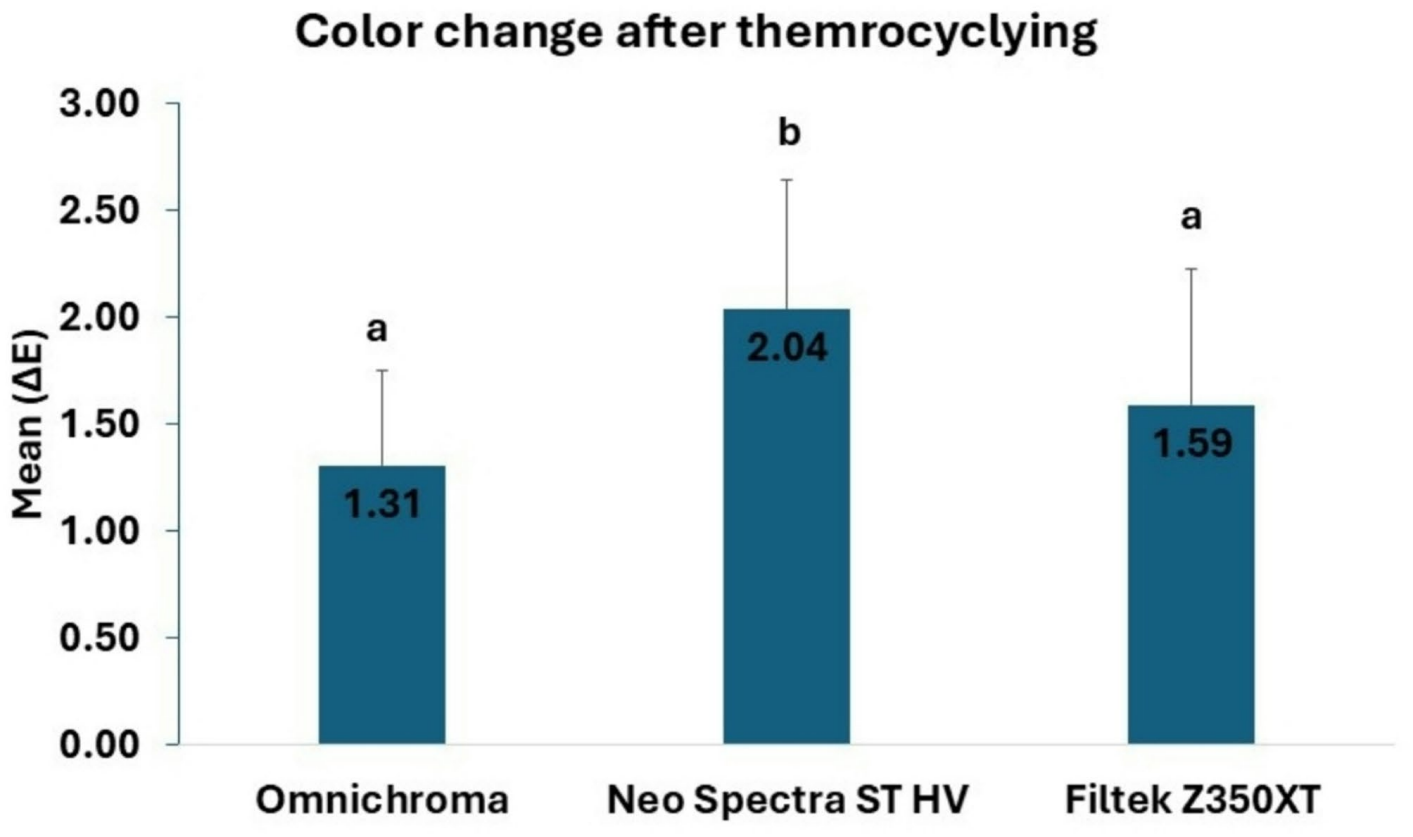
Color change among the three materials different superscript lowercase letters denote statistically significant differences between materials

The color stability of the restorative materials after thermocycling was significantly different (Fig. 1). Group A (Omnichroma) presented the lowest mean color change, which was significantly less than that of Group B (Neo Spectra ST HV) (*p* < 0.001). Furthermore, the mean color change for Group A was less than that for Group C (Filtek Z350XT); however, the difference was not statistically significant (*p* = 0.106). Group B presented the most significant color change, which was much greater than that of Group C (*p* < 0.001).

The findings from the tables provide a comprehensive analysis of the influence of immersion solution and duration on the color stability (∆E) of the composite materials. Across all immersion solutions, Group B Neo Spectra ST HV consistently exhibited the greatest color change, particularly in tea and coffee, compared with both Group A Omnichroma and Group C Filtek Z350XT, with statistically significant differences observed at all time intervals (*p* < 0.001). The findings demonstrate that water immersion had a minimal influence on the color change (∆E) of the examined composite materials at all periods (T1, T2, and T3). At 30 days (T3), the ∆E values for water immersion were not significantly different across the groups (*p* = 0.327). Furthermore, after 30 days of submergence, Group B Neo Spectra ST HV presented significantly greater ∆E values than Group C Filtek Z350XT and Group A Omnichroma (*p* < 0.001). The results indicate that a progressive color change (∆E) occurred with prolonged immersion in tea and coffee among all the composite materials. [Table 2] Two-way repeated-measures ANOVA revealed that all variables—composite material, immersion solution, and immersion duration—significantly affected color change (*p* < 0.001). The immersion period had the most significant influence (partial eta squared = 0.905), followed by the immersion solution (partial eta squared = 0.840) [Table 3].

**Table 2:** Comparison of color changes (∆E) among the research groups after immersion in various staining solutions for different time intervals

Time	Immersion solution	Group A Omnichroma (*n* = 11)	Group B Neo Spectra ST HV (*n* = 11)	Group C Filtek Z350XT (*n* = 11)		*p* value
		Mean ± SD				
T1	Water	2.04 ± 0.93^A^	2.80 ± 1.14^A^	2.77 ± 0.70^A^		0.115
	Tea	3.55 ± 0.72^aB^	7.75 ± 2.03^bB^	4.11 ± 0.82^aB^		< 0.001*
	Coffee	4.55 ± 0.92^aB^	9.15 ± 1.34^bC^	7.25 ± 1.26^cC^		< 0.001*
	***p*** **value**	< 0.001*	< 0.001*	< 0.001*		
T2	Water	2.34 ± 1.22^A^	2.41 ± 1.22^A^	3.24 ± 0.80^A^		0.119
	Tea	5.06 ± 0.91^aB^	9.87 ± 2.75^bB^	6.15 ± 1.17^aB^		< 0.001*
	Coffee	6.37 ± 0.87^aB^	14.27 ± 2.25^bC^	11.32 ± 1.90^cC^		< 0.001*
	***p*** **value**	< 0.001*	< 0.001*	< 0.001*		
T3	Water	2.39 ± 1.38^A^	3.30 ± 2.02^A^	3.23 ± 1.09^A^		0.327
	Tea	7.30 ± 1.28^aB^	11.11 ± 2.93^bB^	8.07 ± 1.36^aB^		< 0.001*
	Coffee	9.99 ± 1.45^aB^	18.22 ± 2.48^bC^	14.48 ± 2.03^cC^		< 0.001*
	***p*** **value**	< 0.001*	< 0.001*	< 0.001*		

T1: immersion for 7 days, T2: immersion for 15 days, T3: immersion for 30 days. (a, b, c) Different superscript lowercase letters denote statistically significant differences between materials. (A, B, C) Different superscript uppercase letters denote statistically significant differences between immersion solutions; SD: standard deviation. ∆E: Delta E (Color difference), n: sample size per group

**Table 3:** Two-way repeated-measures ANOVA for evaluating the impact of the composite material

Parameters	Mean Square	F test	*p* value	Partial Eta Squared
Materials	380.23	57.93	< 0.001*	0.56
Immersion Solutions	1,546.96	235.67	< 0.001*	0.84
Immersion time	462.91	853.51	< 0.001*	0.940
Materials x immersion solutions	96.49	14.70	< 0.001*	0.39
Immersion time x solutions	124.69	229.90	< 0.001*	0.84
Materials x immersion time	5.10	9.41	< 0.001*	0.17
Materials x immersion solutions x immersion time	6.10	11.24	< 0.001*	0.33

*Statistically significant difference with p-value < 0.05. F test: ANOVA test. F test: analysis of variance (ANOVA) test, ∆E: Delta E (Color difference)

### Surface roughness analysis

The surface roughness measurements revealed statistically significant differences among the composite materials before and after thermocycling. Compared with Group B Neo Spectra ST HV (*p* = 0.011) and Group C Filtek Z350XT (*p* < 0.001), Group A Omnichroma had the lowest mean Ra value before thermocycling. After thermocycling, the Ra of Group A Omnichroma slightly increased but remained significantly lower than those of both Group B Neo Spectra ST HV (*p* = 0.026) and Group C Filtek Z350XT (*p* = 0.024). (Table 4)

**Table 4:** Comparison of surface roughness (µm) between the research groups before and after thermocycling

Thermocycling	Group A Omnichroma (*n* = 11)	Group B Neo Spectra ST HV (*n* = 11)	Group C Filtek Z350XT (*n* = 11)	*p* value^1^
	Mean ± SD			
Before	0.13 ± 0.01^a^	0.18 ± 0.04^b^	0.20 ± 0.04^b^	< 0.001*
After	0.16 ± 0.05^a^	0.21 ± 0.03^b^	0.21 ± 0.04^b^	0.012*
*p* value^2^	0.075	0.073	0.540	

*Statistically significant difference p-value < 0.05; *p-value 1*: one-way ANOVA test; *p-value 2*: paired t-test. (a, b) Different superscript lowercase letters denote statistically significant differences between materials. SD: standard deviation. n: sample size per group

After 30 days of immersion in the staining solutions, the surface roughness (Ra) increased with coffee, causing the most significant roughness changes, followed by tea and water. Group A Omnichroma consistently presented the lowest Ra values when immersed in water across all the solutions, which were significantly lower than those of Group B Neo Spectra ST HV (*p* < 0.001*) and Group C Filtek Z350XT (*p* = 0.002*). Compared with both Group A Omnichroma (*p* < 0.001*) and Group C Filtek Z350XT (*p* = 0.032*), Group B Neo Spectra ST HV presented the highest roughness values, particularly after coffee immersion, with significant differences. (Table 5) Two-way ANOVA confirmed significant main effects of both material type (*p* < 0.001, partial eta squared = 0.468) and immersion solution (*p* < 0.001, partial eta squared = 0.328) on Ra but no significant interaction across these factors (*p* = 0.574).

**Table 5:** Comparison of surface roughness (µm) among the studies after immersion in staining solutions

	Group A Omnichroma (*n* = 11)	Group B Neo Spectra ST HV (*n* = 11)	Group C Filtek Z350XT (*n* = 11)
	Mean ± SD		
Water	0.16 ± 0.02^a^	0.22 ± 0.03^b^	0.21 ± 0.04^b^
Tea	0.19 ± 0.01^a^	0.25 ± 0.03^b^	0.23 ± 0.04^b^
Coffee	0.21 ± 0.02^a^	0.28 ± 0.02^b^	0.25 ± 0.03^c^
*Tukey’s post hoc*	P1 = 0.013*, P2 < 0.001*. P3 = 0.029*	P1 = 0.005*, P2 < 0.001*. P3 = 0.098	-

* Statistically significant differences at *p* < 0.05: P1: comparison of water and tea; P2: comparison of water and coffee; P3: comparison of tea and coffee. (a, b, c) Different superscript lowercase letters denote statistically significant differences between materials; SD: standard deviation. n: sample size per group

## Discussion

Determining a tooth’s natural color is the most critical and challenging aspect of restorative dentistry. Nevertheless, achieving a color match of the resin composite to the surrounding tooth remains challenging owing to several influencing variables, including tooth type, location, and age. A recently developed single-shade resin composite (Omnichroma) has been established to match all Vita Classical shades from A1 to D4, diminishing the reliance on therapists’ color-matching skills and facilitating accelerated patient treatment [24].

Omnichroma is free of pigments and utilizes round and uniform supra-nano spherical fillers consisting of silicon dioxide and zirconium dioxide. These fillers create a red-to-yellow fundamental color when light transmits through, reflecting the red-to-yellow spectrum present in all teeth. These colorants subsequently merge with the tooth color, facilitating a successful match. Therefore, the cured composite seamlessly incorporates the adjacent dental structure [25].

Assessment of color change can be conducted via instrumental techniques. In our study, a spectrophotometer with a black background [26] was used for color measurements. This method is ideal for simulating oral environments. While a white background serves as the standard, recent research suggests that a black background is more suitable for evaluating anterior teeth. The black background is designed to replicate light reflectance in restorations that are not surrounded by objects, whereas the white background is designed to represent light reflectance in restorations that are surrounded by objects [27].

In the present study, the impact of thermocycling and staining solutions on the color stability and surface roughness of three different resin composites was evaluated: a supraspherical composite (Omnichroma), a nanohybrid composite (Neo Spectra ST HV), and a nanofilled composite (Filtek Z350XT). The null hypothesis in this study was rejected because of statistically significant changes in the color and surface roughness of all the resin composites before and after thermocycling and staining, as assessed via the Vita Easy Shade and profilometer.

The surface roughness of resin composites often influences the potential for composite discoloration, dental plaque retention, and gingival discomfort. Ideally, the surface roughness of restorations should not exceed 0.2 μm. However, the tongue tip may have a variation in surface roughness of 0.3 μm [28].

In the present study, the average roughness (Ra) values of the resin composite surfaces in all the test groups were below the critical value. Consequently, the results revealed significant differences among the composite materials before and after thermocycling; Omnichroma, in comparison with Neo Spectra ST HV and Filtek Z350XT, had the lowest mean Ra value surface roughness. The size of fillers in composite resins influences the surface roughness of restorations [29]. This may explain the reduced roughness observed in Omnichroma, which is formulated with supranano-filled spherical fillers. In contrast, Neo Spectra nanohybrid composites contain variations in particle size, which may influence the surface texture [30]. On the other hand, Filtek Z350XT nanofilled composition nanoclusters, which are partially calcined irregular porous structures that are not individually silanized, resulted in more water and pigment infiltration [31].

After thermocycling, all the tested composite samples presented increased surface roughness and color changes. These findings are inconsistent with those of previous.

studies [32], which indicated that thermocycling for 10,000 cycles did not significantly affect the surface roughness of the examined resin composites. Similarly [33], reported that a significant difference in the surface roughness of samples subjected to 1000 cycles was detected. These findings could be explained by the fact that thermocycling impacts the mechanical characteristics of composites because of hydrolysis and collapse at the matrix‒resin interface. Moreover, thermocycling has been shown to accelerate the surface deterioration of resin composites by inducing surface stresses and facilitating the production of microcracks [34]. Additionally, water-induced deterioration of the silane interface occurs due to a condensation reaction between the silanol groups of the silica surface and the silane coupling agent, forming siloxane bridge bonds and covalent bonds with the resin components. The siloxane bonds are subsequently degraded by water infiltration into the material, resulting in a hydrolysis process that converts them to a silanol group and triggers debonding of the filler at the interface [35].

Omnichroma revealed the lowest color change in the current study among the composite materials after thermocycling. This can be attributed to Omnichroma’s structural coloring technology, with a significantly improved siloxane bonding technique at the filler and base resin interface. The bonding between the filler and the base resin was identified as significant. Suppose that the interface between the filler and base resin is adequate. In that case, there will be no breakdown caused by hydrolysis of the siloxane link, nor will there be dye infiltration into the filler voids, leading to a reduced susceptibility to discoloration [36].

After immersion in the staining solutions, significant differences in color change and surface roughness were observed in all the tested samples, with the greatest color change and surface roughness in the samples submerged in coffee.

In this study, Omnichroma exhibited the least effect from immersion among the three composite materials. The hydrophobicity of the monomer is an essential factor in predicting the water sorption of a composite resin [37]. Several studies [38, 39] indicate that the existence of TEGDMA in materials results in a much greater hydrophilic capacity and an increased perception of the tonality, water absorption, and stain resistance of Bis-GMA than UDMA. The resin matrix of Omnichroma in this study consists mainly of UDMA, which offers enhanced flexibility and a greater degree of polymerization, leading to superior cross-linking in comparison to the Bis-GMA and TEGDMA monomers found in the resin matrix of the Filtek Z350 XT. This aligns with the findings of another study, which revealed that Omnichroma was least influenced by immersion, which has UDMA in its composition, resulting in lower water sorption than BisGMA and TEGDMA, which are present in the Estalite Palfique LX5 composite resin, resulting in greater roughness in tea and distilled water [40]. These findings contrast with those of a previous study that indicated that Omnichroma exhibited significantly greater color changes, whereas the Filtek Universal composite showed less color change than the Clearfil Majesty ES-2 Premium and Harmonize. This difference is attributed to the presence of the TEGDMA monomer, which is found in all the tested materials except for the Filtek Universal Restorative [41].

Coffee and tea were employed as staining solutions in the present study since they are popular drinks worldwide [42]. To increase the sensitivity of restorations to staining, the staining time was set at 3 h per day since the typical individual requires approximately 60–180 min each day for drinking and eating [43].

The color of the coffee solution used in this study changed more significantly than those of the tea and distilled water solutions, which was in agreement with the findings of numerous studies [44, 45]. This could be attributed to the adsorption and absorption of colorants. The absorption and diffusion of colorants within the organic phase of the materials likely resulted from the attraction between the polymer phase and the yellow colorants in the coffee. The low-polarity yellow stain of the coffee solution penetrates deeper layers of the resin matrix; however, tea, characterized by high-polarity yellow stains, only adsorbs and precipitates on the surface without penetrating the resin matrix [46].

All composite discs in the current study demonstrated significantly increased roughness after immersion in their distinct drinks, with coffee resulting in the highest surface roughness among all the samples. This could be explained by coffee drinking altering the surface roughness via ion precipitation and disturbance of the resin matrix. Moreover, several long-chain organic acids in coffee may solubilize and erode restorative materials, resulting in the surface roughness of composite resins [45]. Similarly, other studies [47, 48] have reported that composite resins exhibit significant solubility in low-pH solutions, and approximately 22 varieties of acids, including citric acid, acetic acid, and malic acid, as well as high-molecular-weight acids, are present in coffee, which exacerbates chemical erosion of material surfaces and increases discoloration.

Omnichroma had the lowest roughness values in this study, whereas the Neo Spectra had the highest roughness after immersion in the staining solutions. This could be explained by the supra-nanofilled Omnichroma composition, which contains a uniform filler particle embedded within the polymer network to minimize the formation of filler-rich and filler-depleted areas that create voids or nonbonding spaces at the filler/matrix interface, which may increase the degree of water sorption in the composite [49]. In contrast, a previous study revealed that the NeoSpectra composite exhibited lower surface roughness because it contains perfectly spherical fillers and is thoroughly impregnated with resin, which is an organically modified ceramic and is indistinguishable from other parts of the filler system [50].

The CIEDE2000 formula is used to evaluate the color difference and perception between two colors. The International Organization for Standardization (ISO) and the International Commission on Illumination (CIE) recommend the use of the CIEDE2000 color difference formula for better visual perception match; for this reason, this formula was used in the present study. Acceptability is related to the human eye’s acceptance of a restoration’s color, whereas perceptibility measures the color difference between two objects [51].

The perceptibility threshold (ΔE00) in this study was established at 0.8. Conversely, the 50:50% acceptability threshold (ΔE00) was 1.8 [15]. In the present study, the perceptibility threshold values for all the groups exceeded 0.8. After 30 days of immersion in the staining solutions, the acceptability threshold values were greater than 1.8 for all groups, except for Group A (Omnichroma), which had a ∆E of 1.31 ± 0.44, and Group C (Filtek Z350XT), which had a ∆E of 1.59 ± 0.64 after thermocycling. The findings align with those of the present study, indicating that the Omnichroma group presented a ΔE1 value of 1.33 ± 0.56, reflecting the color change from baseline to 10 days of simulated staining, brushing, and thermocycling, which remained below the perceptual threshold, indicating that there was no clinically observable color change at this point. These findings indicate that Omnichroma exhibits significant short-term color stability. The ΔE2 values for Omnichroma (9.62 ± 1.39), which reflect the color change from 10 days to 1 year of simulated staining, brushing, and thermocycling, were significantly above both thresholds. This indicates considerable discoloration over time under simulated intraoral conditions [52].

Limitations of the present study include the significance of the matrix composition. Compared with the Bis-GMA-free single-shade composite, the universal composite containing BisGMA exhibited a marked increase in color change and surface roughness. Additional studies are necessary to examine the influence of aging on the long-term effects and degradation associated with surface roughness, which could act as retention sites for plaque, bacteria, or food debris, ultimately influencing the integrity and longevity of dental restorations. Several factors influence restorations in the oral cavity, including saliva flow, microbiota, changes in pH, and fluctuations in temperature. Therefore, additional investigations and in vivo studies are necessary to assess the color-matching capability of single-shaded resin composites within the oral environment [53]. Our study utilized three distinct liquids with three different composites. Nevertheless, additional investigations are needed, including investigations of other liquids and composites with diverse compositions [38]. The employed aging method is valid; nevertheless, future research could investigate alternative aging strategies and longer durations following specimen finishing and polishing and assess their effects. Further research using dynamic immersion procedures is crucial because the strength of staining from each agent leads to continuous exposure since both the staining solutions and specimens were kept statically in the container. However, actual staining in the oral cavity is characterized by the intermittent nature of stain exposure, since saliva and other fluids dilute the staining agents.

## Conclusion

Within the limitations of the current study, the nanofilled resin composite showed more acceptable color stability and roughness values. However, the staining solutions used affected the color stability and surface roughness of the resin composites. Furthermore, coffee has a more pronounced impact than other beverages, particularly on nanohybrid resin composites. Coffee can change the properties of composite materials, leading to variations in color and alterations in surface roughness. The specific material used contributed to variations in surface roughness and color stability.

## Electronic supplementary material

Below is the link to the electronic supplementary material.

Supplementary Material 1

Supplementary Material 2

Supplementary Material 3

Supplementary Material 4

Supplementary Material 5

Supplementary Material 6

Supplementary Material 7

Supplementary Material 8

Supplementary Material 9

## Data Availability

Data is available on request from the corresponding author.

## References

[1] Catelan A, Suzuki TYU, Becker F Jr., Briso ALF, Dos Santos PH. Influence of surface sealing on color stability and roughness of composite submitted to ultraviolet-accelerated aging. J Investig Clin Dent. 2017;8. 10.1111/jicd.12203. 10.1111/jicd.12203 26748677

[2] Uctasli MB, Garoushi S, Uctasli M, Vallittu PK, Lassila L. A comparative assessment of color stability among various commercial resin composites. BMC Oral Health. 2023;23:789. 10.1186/s12903-023-03515-9. 10.1186/s12903-023-03515-9 PMC10598901 37875872

[3] Tekçe N, Tuncer S, Demirci M, Serim ME, Baydemir C. The effect of different drinks on the color stability of different restorative materials after one month. Restor Dent Endod. 2015;40:255–261. 10.5395/rde.2015.40.4.255. 10.5395/rde.2015.40.4.255 PMC4650520 26587410

[4] Igiel C, Lehmann KM, Ghinea R, Weyhrauch M, Hangx Y, Scheller H et al. Reliability of visual and instrumental color matching. J Esthet Restor Dent. 2017;29:303–308. 10.1111/jerd.12321. 10.1111/jerd.12321 28742283

[5] Babina K, Polyakova M, Sokhova I, Doroshina V, Arakelyan M, Novozhilova N. The effect of finishing and Polishing sequences on the surface roughness of three different nanocomposites and composite/enamel and composite/cementum interfaces. Nanomaterials (Basel). 2020;10. 10.3390/nano10071339. 10.3390/nano10071339 PMC7407209 32659992

[6] Nawrocka A, Piwonski I, Sauro S, Porcelli A, Hardan L, Lukomska-Szymanska M. Traditional microscopic techniques employed in dental adhesion Research-Applications and protocols of specimen Preparation. Biosens (Basel). 2021;11. 10.3390/bios11110408. 10.3390/bios11110408 PMC8615842 34821624

[7] Ahmed MA, Jouhar R, Khurshid Z. Smart Monochromatic Composite: A Literature Review. Int J Dent. 2022;2022:2445394. 10.1155/2022/2445394. 10.1155/2022/2445394 PMC9666026 36398065

[8] Alfawaz Y. Impact of Polishing systems on the surface roughness and microhardness of nanocomposites. J Contemp Dent Pract. 2017;18:647–51. 10.5005/jp-journals-10024-2100. 28816183 10.5005/jp-journals-10024-2100

[9] Sirin Karaarslan E, Bulbul M, Yildiz E, Secilmis A, Sari F, Usumez A. Effects of different polishing methods on color stability of resin composites after accelerated aging. Dent Mater J. 2013;32:58–67. 10.4012/dmj.2012-045. 10.4012/dmj.2012-045 23370871

[10] Gupta S, Dhawan R. The effect of various finishing and Polishing systems on the surface roughness of four composite resin Materials–An in vitro study. J Contemp Dent. 2012;3.

[11] Daud A, Gray G, Lynch CD, Wilson NHF, Blum IR. A randomised controlled study on the use of finishing and polishing systems on different resin composites using 3D contact optical profilometry and scanning electron microscopy. J Dent. 2018;71:25–30. 10.1016/j.jdent.2018.01.008. 10.1016/j.jdent.2018.01.008 29360492

[12] Kritzinger D, Brandt P, De Wet F. The effect of different Polishing systems on the surface roughness of a nanocomposite and a microhybrid composite. S Afr Dent J. 2017;72:249–57.

[13] Ishii R, Takamizawa T, Tsujimoto A, Suzuki S, Imai A, Barkmeier WW et al. Effects of Finishing and Polishing Methods on the Surface Roughness and Surface Free Energy of Bulk-fill Resin Composites. Oper Dent. 2020;45:E91-e104. 10.2341/18-246-l. 10.2341/18-246-L 31738697

[14] Schmitt VL, Puppin-Rontani RM, Naufel FS, Nahsan FP, Alexandre Coelho Sinhoreti M, Baseggio W. Effect of the polishing procedures on color stability and surface roughness of composite resins. ISRN Dent. 2011;2011:617672. 10.5402/2011/617672. 10.5402/2011/617672 PMC3169916 21991483

[15] Rohym S, Tawfeek HEM, Kamh R. Effect of coffee on color stability and surface roughness of newly introduced single shade resin composite materials. BMC Oral Health. 2023;23:236. 10.1186/s12903-023-02942-y. 10.1186/s12903-023-02942-y PMC10122801 37087507

[16] Sharma G, Wu W, Dalal EN. The CIEDE2000 color-difference formula: implementation notes, supplementary test data, and mathematical observations. Color Res Application. 2005;30:21–30. 10.1002/col.20070.

[17] Taşın S, Ismatullaev A, Usumez A. Comparison of surface roughness and color stainability of 3-dimensionally printed interim prosthodontic material with conventionally fabricated and CAD-CAM milled materials. J Prosthet Dent. 2022;128:1094–101. 10.1016/j.prosdent.2021.01.027. 33715836 10.1016/j.prosdent.2021.01.027

[18] Toledano M, Osorio E, Osorio R, García-Godoy F. Microleakage of class V resin-modified glass ionomer and compomer restorations. J Prosthet Dent. 1999;81:610–5. 10.1016/s0022-3913(99)70217-9. 10220667 10.1016/s0022-3913(99)70217-9

[19] Shetty P, Purayil TP, Ginjupalli K, Pentapati KC. Effect of polishing technique and immersion in beverages on color stability of nanoceramic composites. J Oral Biol Craniofac Res. 2021;11:53–56. 10.1016/j.jobcr.2020.11.011. 10.1016/j.jobcr.2020.11.011 PMC7736987 33344162

[20] Arocha MA, Mayoral JR, Lefever D, Mercade M, Basilio J, Roig M. Color stability of siloranes versus methacrylate-based composites after immersion in staining solutions. Clin Oral Investig. 2013;17:1481–1487. 10.1007/s00784-012-0837-7. 10.1007/s00784-012-0837-7 22993112

[21] Polli MJ, Arossi GA. Effect of finishing and Polishing on the color stability of a composite resin immersed in staining solutions. J Dent Res. 2015;2:120–6.

[22] Barutcigil Ç, Yıldız M. Intrinsic and extrinsic discoloration of dimethacrylate and Silorane based composites. J Dent. 2012;40(Suppl 1):e57–63. 10.1016/j.jdent.2011.12.017. 22239912 10.1016/j.jdent.2011.12.017

[23] Koc-Vural U, Baltacioglu I, Altinci P. Color stability of bulk-fill and incremental-fill resin-based composites polished with aluminum-oxide impregnated disks. Restor Dent Endod. 2017;42:118–124. 10.5395/rde.2017.42.2.118. 10.5395/rde.2017.42.2.118 PMC5426223 28503477

[24] Kobayashi S, Nakajima M, Furusawa K, Tichy A, Hosaka K, Tagami J. Color adjustment potential of single-shade resin composite to various-shade human teeth: Effect of structural color phenomenon. Dent Mater J. 2021;40:1033–1040. 10.4012/dmj.2020-364. 10.4012/dmj.2020-364 33883353

[25] Ebaya MM, Ali AI, El-Haliem HA, Mahmoud SH. Color stability and surface roughness of ormocer- versus methacrylate-based single shade composite in anterior restoration. BMC Oral Health. 2022;22:430. 10.1186/s12903-022-02423-8. 10.1186/s12903-022-02423-8 PMC9513900 36167560

[26] El-Rashidy AA, Abdelraouf RM, Habib NA. Effect of two artificial aging protocols on color and gloss of single-shade versus multi-shade resin composites. BMC Oral Health. 2022;22:321. 10.1186/s12903-022-02351-7. 35915423 10.1186/s12903-022-02351-7 PMC9341039

[27] Kang YA, Lee HA, Chang J, Moon W, Chung SH, Lim BS. Color Stability of Dental Reinforced CAD/CAM Hybrid Composite Blocks Compared to Regular Blocks. Materials (Basel). 2020;13. 10.3390/ma13214722. 10.3390/ma13214722 PMC7660196 33105868

[28] Abdelaziz KM, Mir S, Khateeb SU, Baba SM, Alshahrani SS, Alshahrani EA et al. Influences of successive exposure to bleaching and fluoride preparations on the surface hardness and roughness of the aged resin composite restoratives. Medicina (Kaunas). 2020;56:1–12. 10.3390/medicina56090476. 10.3390/medicina56090476 PMC7557809 32947937

[29] Altınışık H, Özyurt E. Effect of different Polishing systems on surface roughness and gloss values of single-shade resin composites. BMC Oral Health. 2024;24(1391). 10.1186/s12903-024-05163-z. 10.1186/s12903-024-05163-z PMC11568551 39548450

[30] Turssi CP, Ferracane JL, Ferracane LL. Wear and fatigue behavior of nano-structured dental resin composites. J Biomed Mater Res B Appl Biomater. 2006;78:196–203. 10.1002/jbm.b.30475. 16447169 10.1002/jbm.b.30475

[31] Hashemikamangar SS, Farahani S, Khoshgoo S, Doroudgar P. Comparative Efficacy of Four Stain Removal Methods for Bleach-Shade Composite Resins after Immersion in Staining Solutions: An In Vitro Study. Int J Dent. 2023;2023:8909288. 10.1155/2023/8909288. 10.1155/2023/8909288 PMC10277192 37342250

[32] Tuncer S, Demirci M, Tiryaki M, Unlü N, Uysal Ö. The effect of a modeling resin and thermocycling on the surface hardness, roughness, and color of different resin composites. J Esthet Restor Dent. 2013;25:404–19. 10.1111/jerd.12063. 24172016 10.1111/jerd.12063

[33] Imtiaz T, Ganesh SB, Jayalakshmi S. Surface roughness changes of two composite resin restorative materials after thermocycling. J Adv Pharm Technol Res. 2022;13(S466–s469). 10.4103/japtr.japtr_255_22. 10.4103/japtr.japtr_255_22 PMC9926603 36798583

[34] El-Rashidy AA, Shaalan O, Abdelraouf RM, Habib NA. Effect of immersion and thermocycling in different beverages on the surface roughness of single- and multi-shade resin composites. BMC Oral Health. 2023;23:367. 10.1186/s12903-023-03069-w. 10.1186/s12903-023-03069-w PMC10249292 37287027

[35] Curtis AR, Shortall AC, Marquis PM, Palin WM. Water uptake and strength characteristics of a nanofilled resin-based composite. J Dent. 2008;36:186–193. 10.1016/j.jdent.2007.11.015. 10.1016/j.jdent.2007.11.015 18237839

[36] Maesako M, Kishimoto T, Tomoda S, Horie T, Yamada M, Iwawaki R et al. Evaluation of the Repolished Surface Properties of a Resin Composite Employing Structural Coloration Technology. Materials (Basel). 2021;14. 10.3390/ma14237280. 10.3390/ma14237280 PMC8658579 34885437

[37] Szczesio-Wlodarczyk A, Domarecka M, Kopacz K, Sokolowski J, Bociong K. An evaluation of the properties of urethane Dimethacrylate-Based dental resins. Mater (Basel). 2021;14. 10.3390/ma14112727. 10.3390/ma14112727 PMC8196897 34064213

[38] Sensi L, Winkler C, Geraldeli S. Accelerated aging effects on color stability of potentially color adjusting Resin-based composites. Oper Dent. 2021;46:188–96. 10.2341/20-099-l. 34086953 10.2341/20-099-L

[39] Ortengren U, Wellendorf H, Karlsson S, Ruyter IE. Water sorption and solubility of dental composites and identification of monomers released in an aqueous environment. J Oral Rehabil. 2001;28:1106–15. 10.1046/j.1365-2842.2001.00802.x. 11874509 10.1046/j.1365-2842.2001.00802.x

[40] Alshehri A, Alhalabi F, Mustafa M, Awad MM, Alqhtani M, Almutairi M et al. Effects of Accelerated Aging on Color Stability and Surface Roughness of a Biomimetic Composite: An In Vitro Study. Biomimetics (Basel). 2022;7. 10.3390/biomimetics7040158. 10.3390/biomimetics7040158 PMC9624352 36278715

[41] Aydın N, Karaoğlanoğlu S, Oktay EA, Ersöz B. Investigation of single shade composite resin surface roughness and color stability. Atatürk Üniversitesi Diş Hekimliği Fakültesi Dergisi. 2021;31:207–214. 10.17567/ataunidfd.895734.

[42] Alkhadim YK, Hulbah MJ, Nassar HM. Color Shift, Color Stability, and Post-Polishing Surface Roughness of Esthetic Resin Composites. Materials (Basel). 2020;13. 10.3390/ma13061376. 10.3390/ma13061376 PMC7143460 32197532

[43] Hussain SK, Al-Abbasi SW, Refaat MM, Hussain AM. The effect of staining and bleaching on the color of two different types of composite restoration. J Clin Exp Dent. 2021;13:e1233-e1238. 10.4317/jced.58837. 10.4317/jced.58837 PMC8715564 34987716

[44] Mara da Silva T, Barbosa Dantas DC, Franco TT, Franco LT, Rocha Lima Huhtala MF. Surface degradation of composite resins under staining and brushing challenges. J Dent Sci. 2019;14:87–92. 10.1016/j.jds.2018.11.005. 10.1016/j.jds.2018.11.005 PMC6445979 30988884

[45] Cumhur A, Özkoçak BBC. Evaluation of the color stability and surface roughness of a novel single-shade composite resin: A smart chromatic technology. Cyprus J Med Sci. 2024;9:28–35. 10.4274/cjms.2023.2023-37.

[46] Reddy PS, Tejaswi KL, Shetty S, Annapoorna BM, Pujari SC, Thippeswamy HM. Effects of commonly consumed beverages on surface roughness and color stability of the nano, microhybrid and hybrid composite resins: an in vitro study. J Contemp Dent Pract. 2013;14:718–723. 10.5005/jp-journals-10024-1390. 10.5005/jp-journals-10024-1390 24309354

[47] Tian F, Yap AU, Wang X, Gao X. Effect of staining solutions on color of pre-reacted glass-ionomer containing composites. Dent Mater J. 2012;31:384–388. 10.4012/dmj.2011-223. 10.4012/dmj.2011-223 22673467

[48] Tan BL, Yap AU, Ma HN, Chew J, Tan WJ. Effect of beverages on color and translucency of new tooth-colored restoratives. Oper Dent. 2015;40:E56-65. 10.2341/149027-l. 10.2341/149027-L 25275960

[49] Festuccia MS, Garcia Lda F, Cruvinel DR, Pires-De-Souza Fde C. Color stability, surface roughness and microhardness of composites submitted to mouthrinsing action. J Appl Oral Sci. 2012;20:200–205. 10.1590/s1678-77572012000200013. 10.1590/S1678-77572012000200013 PMC3894763 22666837

[50] Redwan HS, Hussein MA, Abdul-Monem MM. Effect of Bleaching on Surface Roughness and Color Parameters of Coffee-Stained Nanohybrid Dental Composites with Different Viscosities. Eur J Gen Dent. 2024;14:027–035. 10.1055/s-0044-1786752.

[51] Durand LB, Ruiz-López J, Perez BG, Ionescu AM, Carrillo-Pérez F, Ghinea R et al. Color, lightness, chroma, hue, and translucency adjustment potential of resin composites using CIEDE2000 color difference formula. J Esthet Restor Dent. 2021;33:836–843. 10.1111/jerd.12689. 10.1111/jerd.12689 33283966

[52] Tepe H, Celiksoz O, Yaman BC. Evaluation of color stability in single-shade composite resins using spectrophotometer and cross-polarized mobile photography. BMC Oral Health. 2025;25:280. 10.1186/s12903-025-05651-w. 10.1186/s12903-025-05651-w PMC11846188 39987109

[53] Engel AS, Kranz HT, Schneider M, Tietze JP, Piwowarcyk A, Kuzius T et al. Biofilm formation on different dental restorative materials in the oral cavity. BMC Oral Health. 2020;20:162. 10.1186/s12903-020-01147-x. 10.1186/s12903-020-01147-x PMC7268681 32493365

