# Supplementary figures and images for: Evaluation of color stability and surface roughness of smart monochromatic resin composite in comparison to universal resin composites after immersion in staining solutions
Source: BMC Oral Health. 2025 Jul 19;25:1211. doi: 10.1186/s12903-025-06555-5 (PMC12276654; doi:10.1186/s12903-025-06555-5)

| 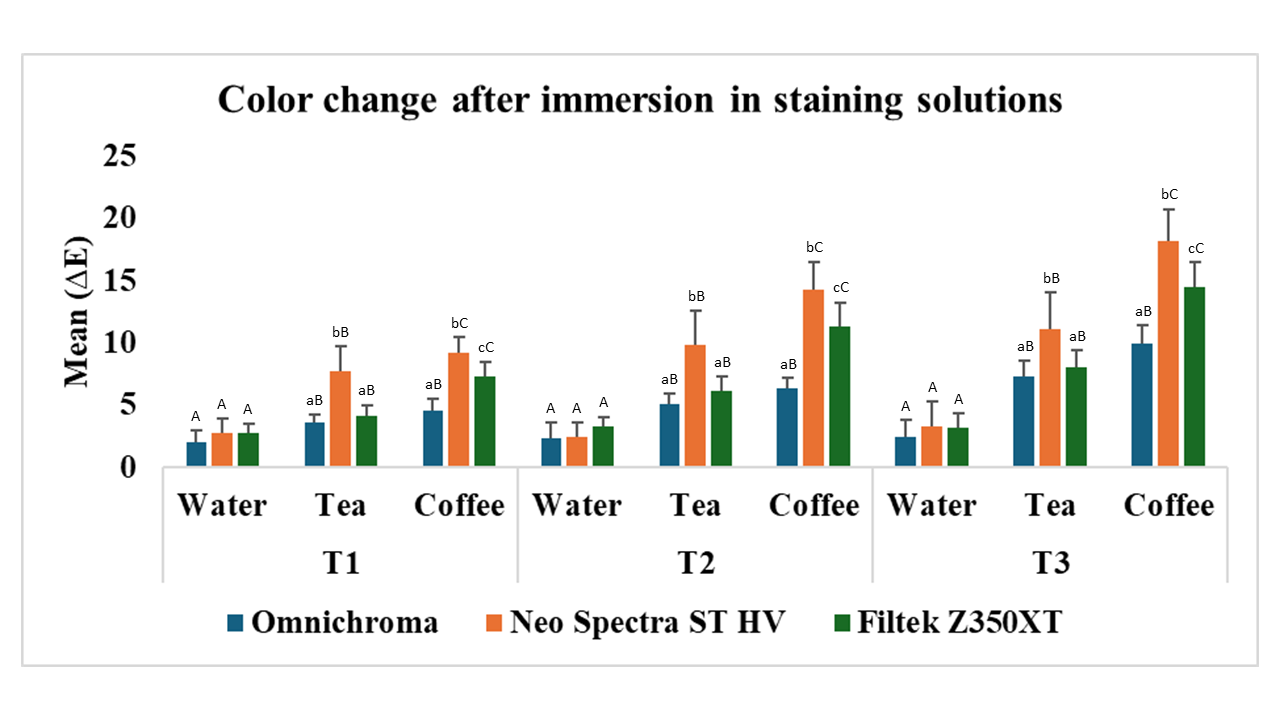 |
| --- |
|  |

Supplement: Supplementary file 1 — Supplementary Material 1 [file 12903_2025_6555_MOESM1_ESM.docx]

| 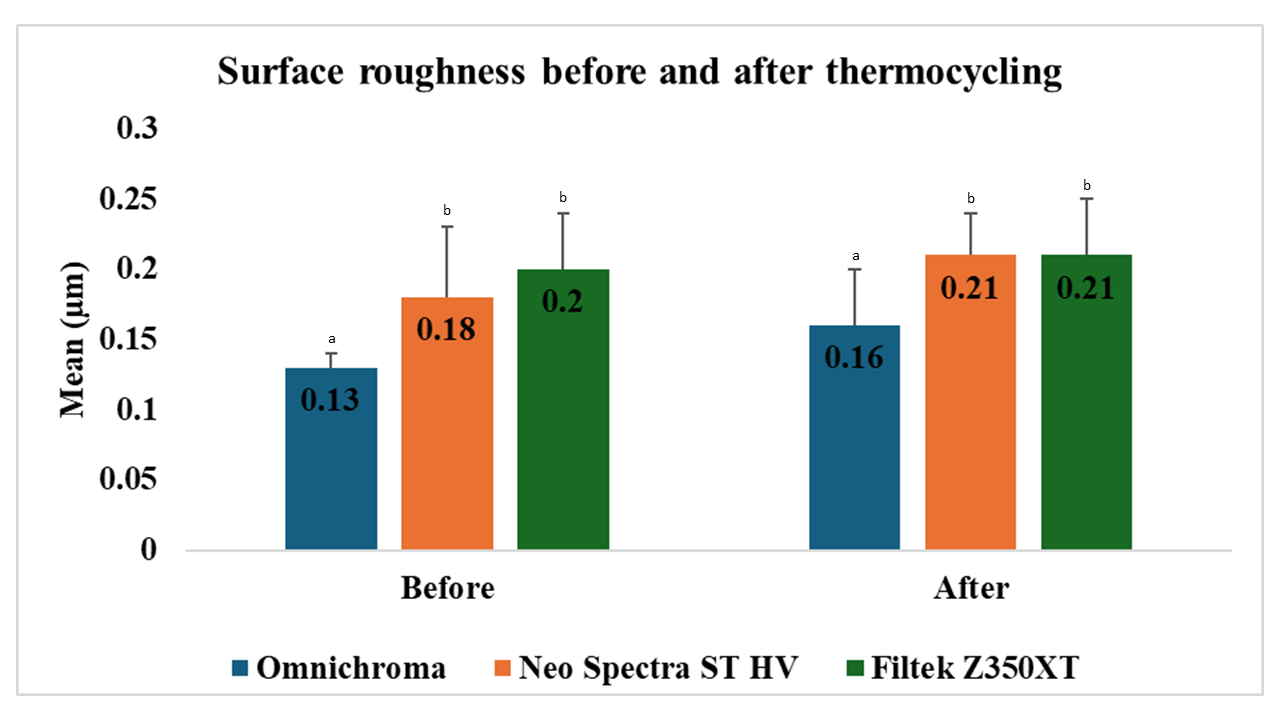 |
| --- |
|  |

Supplement: Supplementary file 2 — Supplementary Material 2 [file 12903_2025_6555_MOESM2_ESM.docx]

| 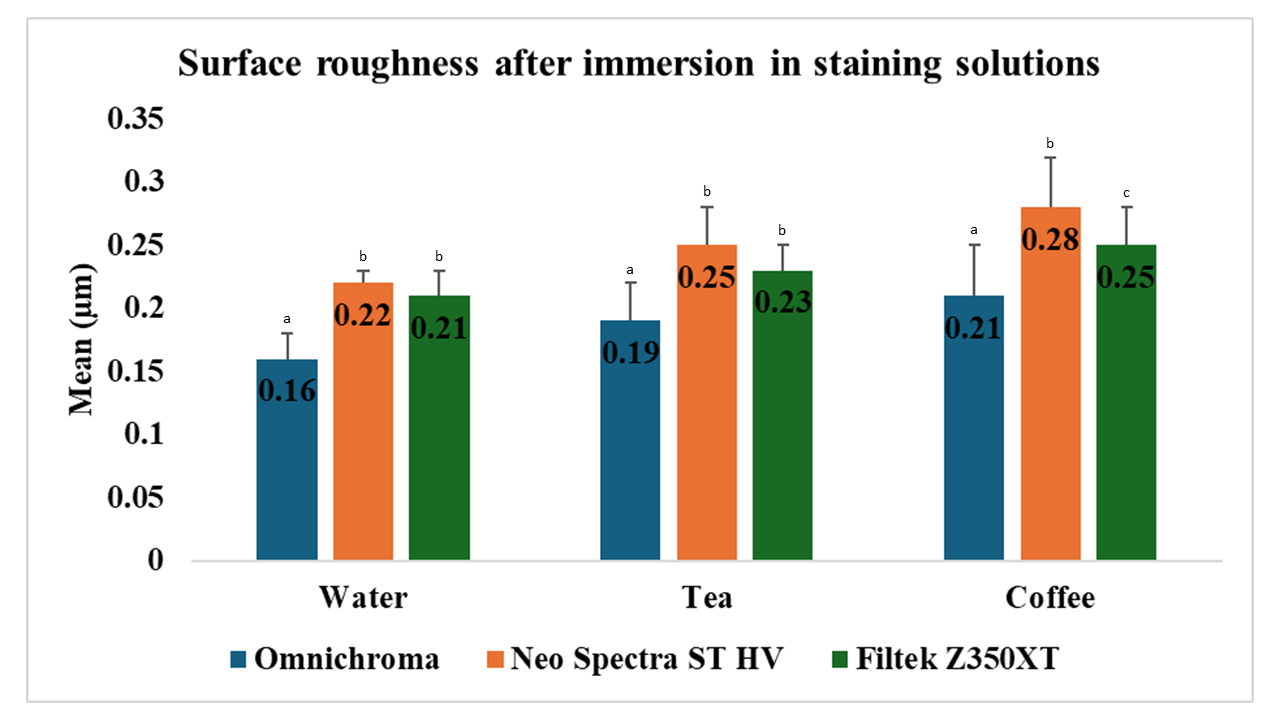 |
| --- |
|  |

Supplement: Supplementary file 3 — Supplementary Material 3 [file 12903_2025_6555_MOESM3_ESM.docx]
