# Supplementary material for: Evaluation of color stability and surface roughness of smart monochromatic resin composite in comparison to universal resin composites after immersion in staining solutions
Source: BMC Oral Health. 2025 Jul 19;25:1211. doi: 10.1186/s12903-025-06555-5 (PMC12276654; doi:10.1186/s12903-025-06555-5)
Supplement: Supplementary file 4 — Supplementary Material 4 [file 12903_2025_6555_MOESM4_ESM.docx]

**Table A: Comparison of the color change (∆E) among the study groups after thermocycling**

|  | Group A  Omnichroma  (n=11) | Group B  Neo Spectra ST HV  (n=11) | Group C  Filtek Z350XT  (n=11) | |
| --- | --- | --- | --- | --- |
|  | Mean ±SD | | |  |
| ∆E | 1.31 ±0.44 | 2.04 ±0.60 | 1.59 ±0.64 | |
| *p value 1* | <0.001* | | | |
| Tukey’s post-hoc | P1<0.001*, P2=0.106, P3<0.001* | | | |

*Statistically significant difference at p value < 0.05, p value 1: One-Way ANOVA test, P1: comparison between Omnichroma and Neo Spectra ST HV, P2: comparison between Omnichroma and Filtek Z350XT, P3: comparison between Neo Spectra ST HV and Filtek Z350XT
