# Supplementary material for: Evaluation of color stability and surface roughness of smart monochromatic resin composite in comparison to universal resin composites after immersion in staining solutions
Source: BMC Oral Health. 2025 Jul 19;25:1211. doi: 10.1186/s12903-025-06555-5 (PMC12276654; doi:10.1186/s12903-025-06555-5)
Supplement: Supplementary file 5 — Supplementary Material 5 [file 12903_2025_6555_MOESM5_ESM.docx]

**Table B: Pairwise comparison regarding the color change (∆E) among different materials for each immersion solution with different immersion times**

| Immersion solution | Groups | Compared to | *P-value* | | |
| --- | --- | --- | --- | --- | --- |
|  |  |  | T1 | T2 | T3 |
| Tea | Omnichroma | Neo Spectra ST HV | <0.001* | <0.001* | <0.001* |
|  |  | Filtek Z350XT | 0.590 | 0.345 | 0.642 |
|  | Neo Spectra ST HV | Filtek Z350XT | <0.001* | <0.001* | 0.004* |
| Coffee | Omnichroma | Neo Spectra ST HV | <0.001* | <0.001* | <0.001* |
|  |  | Filtek Z350XT | <0.001* | <0.001* | <0.001* |
|  | Neo Spectra ST HV | Filtek Z350XT | 0.002* | 0.001* | <0.001* |

*Statistically significant difference at p value < 0.05, T1: immersion for 7 days, T2: immersion for 15 days, T3: immersion for 30 days
