# Supplementary material for: Evaluation of color stability and surface roughness of smart monochromatic resin composite in comparison to universal resin composites after immersion in staining solutions
Source: BMC Oral Health. 2025 Jul 19;25:1211. doi: 10.1186/s12903-025-06555-5 (PMC12276654; doi:10.1186/s12903-025-06555-5)
Supplement: Supplementary file 6 — Supplementary Material 6 [file 12903_2025_6555_MOESM6_ESM.docx]

**Table C: Pairwise comparison regarding the color change (∆E) among different immersion solutions for each material at different immersion times**

| Materials | Groups | Compared to | *P-value* | | |
| --- | --- | --- | --- | --- | --- |
|  |  |  | T1 | T2 | T3 |
| Omnichroma | Water | Tea | 0.001* | <0.001* | <0.001* |
|  |  | Coffee | <0.001* | <0.001* | <0.001* |
|  | Tea | Coffee | 0.207* | 0.014* | <0.001* |
| Neo Spectra ST HV | Water | Tea | <0.001* | <0.001* | <0.001* |
|  |  | Coffee | <0.001* | <0.001* | <0.001* |
|  | Tea | Coffee | 0.105 | <0.001* | <0.001* |
| Filtek Z350XT | Water | Tea | 0.007* | <0.001* | <0.001* |
|  |  | Coffee | <0.001* | <0.001* | <0.001* |
|  | Tea | Coffee | <0.001* | <0.001* | <0.001* |

*Statistically significant difference at p value < 0.05, T1: immersion for 7 days, T2: immersion for 15 days, T3: immersion for 30 days
