# Supplementary material for: Evaluation of color stability and surface roughness of smart monochromatic resin composite in comparison to universal resin composites after immersion in staining solutions
Source: BMC Oral Health. 2025 Jul 19;25:1211. doi: 10.1186/s12903-025-06555-5 (PMC12276654; doi:10.1186/s12903-025-06555-5)
Supplement: Supplementary file 7 — Supplementary Material 7 [file 12903_2025_6555_MOESM7_ESM.docx]

**Table D: Pairwise comparison regarding the surface roughness (µm) among different materials before and after thermocycling**

| Groups | Compared to | *P value 1* | |
| --- | --- | --- | --- |
|  |  | Before | After |
| Omnichroma | Neo Spectra ST HV | 0.011* | 0.026* |
|  | Filtek Z350XT | <0.001* | 0.024* |
| Neo Spectra ST HV | Filtek Z350XT | 0.315 | 1.00 |

*Statistically significant difference at p value < 0.05, P value 1: Tukey’s post hoc test
