# Supplementary material for: Evaluation of color stability and surface roughness of smart monochromatic resin composite in comparison to universal resin composites after immersion in staining solutions
Source: BMC Oral Health. 2025 Jul 19;25:1211. doi: 10.1186/s12903-025-06555-5 (PMC12276654; doi:10.1186/s12903-025-06555-5)
Supplement: Supplementary file 8 — Supplementary Material 8 [file 12903_2025_6555_MOESM8_ESM.docx]

**Table E: Two-Way ANOVA assessing the effect of composite material and aging process (thermocycling and staining solutions) on surface roughness**

|  | Mean Square | F-test | *p-value* | Partial Eta Squared |
| --- | --- | --- | --- | --- |
| Materials | 0.032 | 39.531 | <0.001* | 0.468 |
| Immersion solutions | 0.018 | 21.949 | <0.001* | 0.328 |
| Materials x immersion solutions | 0.001 | 0.730 | 0.574 | 0.031 |

*Statistically significant difference at p value < 0.05, Adjusted R squared = 0.546
