# Supplementary material for: Evaluation of color stability and surface roughness of smart monochromatic resin composite in comparison to universal resin composites after immersion in staining solutions
Source: BMC Oral Health. 2025 Jul 19;25:1211. doi: 10.1186/s12903-025-06555-5 (PMC12276654; doi:10.1186/s12903-025-06555-5)
Supplement: Supplementary file 9 — Supplementary Material 9 [file 12903_2025_6555_MOESM9_ESM.docx]

**Table F: Pairwise comparison of surface roughness between the groups for each immersion solution.**

| Groups | Compared to | *P value 1* | | |
| --- | --- | --- | --- | --- |
|  |  | **Water** | **Tea** | **Coffee** |
| Omnichroma | Neo Spectra ST HV | 0.001* | <0.001* | <0.001* |
|  | Filtek Z350XT | 0.002* | 0.006* | 0.006* |
| Neo Spectra ST HV | Filtek Z350XT | 0.992 | 0.106 | 0.032* |

*Statistically significant difference at p value < 0.05, P value 1: Tukey’s post hoc test
